# Anxiety and depression levels of healthcare workers during Covid-19 pandemic

**DOI:** 10.4314/ahs.v22i1.62

**Published:** 2022-03

**Authors:** Onur Turan, Nilgün Yılmaz Demirci, AK Güntülü, Şule Akçay, Ülkü Aka Aktürk, Semra Bilaçeroğlu, Funda Coşkun, Oğuz Köktürk, Arzu Mirici, Cengiz Özdemir, Nazan Şen, Ülkü Yilmaz

**Affiliations:** 1 Izmir Katip Celebi University Atatürk Research and Training Hospital, Chest Diseases Department, İzmir-Turkey; 2 Gazi University, Chest Diseases Department, Ankara – Turkey; 3 Eskişehir Osmangazi University, Chest Diseases Department, Eskişehir – Turkey; 4 Başkent University, Chest Diseases Department, Ankara – Turkey; 5 Süreyyapaşa Research and Training Hospital, Chest Diseases Department, İstanbul-Turkey; 6 İzmir Dr. Suat Seren Chest Diseases and Chest Surgery Hospital, Chest Diseases Department, İzmir – Turkey; 7 Uludağ University, Chest Diseases Department, Bursa – Turkey; 8 Canakkale 18 Mart University, Chest Diseases Department, Canakkale – Turkey; 9 Yedikule Research and Training Hospital, Chest Diseases Department, İstanbul-Turkey; 10 Başkent University, Turgut Noyan Research and Training Hospital, Chest Diseases Department, Adana-Turkey; 11 Ankara Atatürk Chest Diseases and Chest Surgery Research and Training Hospital, Chest Diseases Department, Ankara-Turkey

**Keywords:** Healthcare workers, anxiety, depression, covid-19

## Abstract

**Background:**

Coronavirus disease 2019 (covid-19), which causes a pandemic in the world, has started to appear in turkey since march 2020. Healthcare workers are at the top of the groups most at risk for covid-19 infection, which can have a negative impact on psychological state.

**Objectives:**

It was aimed to evaluate anxiety and depression levels among healthcare workers.

**Methods:**

this cross-sectional study performed via an online survey in april 2020. Participants answered questions about sociodemographic features, personal views and experiences about covid-19 and the hospital anxiety and depression scale (hads).

**Results:**

A total of 300 healthcare workers,193 men and 107 women, participated in the survey. According to hads, 44.6% of participants scored above anxiety and 68.2% scored above depression cut-off points. Being younger than 50 and taking care of covid-19 patients in hospitals were independently associated with anxiety risk. Female gender, young age (less than 50) and having comorbidity were independent risk factors for depression.

**Conclusion:**

Healthcare workers were at high risk of anxiety and depression during covid-19 outbreak. For this reason, psychological support should be given, especially to the group with high risk.

## Introduction

On December 2019, some cases of pneumonia of unknown etiology occurred in Wuhan City, China. A new type of virus, named as coronavirus disease 2019 (COVID-19), was found to be the cause of pneumonia^1^. Because of rapid and worldwide spread of the disease, the outbreak has been declared as pandemic on March 11, 2020 by WHO. The COVID-19 pandemic was confirmed to have reached Turkey in March 2020. Since then, the number of confirmed total cases in Turkey surpassed 120.000 at the beginning of May 2020^2^.

From doctors to nurses, all medical health workers are first-line fighters treating patients with COVID-19 3. This fact makes healthcare workers to have a high risk of exposure to the virus all around the world. Europe had the highest absolute numbers of reported COVID-19 infections (119 628), and the Eastern Mediterranean region had the highest number of reported deaths per 100 infections among healthcare workers 4. The total number of health workers infected COVID-19 in Turkey has reached 7428 according to ministry of health at the end of April 2020 2.

Anxiety and depression are common in healthcare professionals due to the stressful working environment and long working hours. More than a fifth of healthcare professionals working in the hospitals screened positive for depression or anxiety ahead of the coronavirus pandemic^5^.

Outbreaks like COVID-19 not only injure the health, but also have a negative psychological impact on healthcare workers. Previous studies considered severe acute respiratory syndrome (SARS) 2003 outbreak to be a psychological trauma among frontline healthcare workers^6^. As they are directly involved in the diagnosis and treatment of COVID-19, they also face a risk of developing psychological distress about this situation. However, there is no study targeting the psychological status of healthcare workers in Turkey yet.

There are many factors that may affect the psychological mood in people, such as gender and age. It has been well established that women are in greater risk for anxiety and depression than men, because of the interactions between biological factors and social determinants 7. Older persons have significantly lower frequencies of any current anxiety disorder, but they have been at greater risk of developing depression because of the cumulative effect of numerous risk factors, such as chronic illness and isolation 8. These parameters can also affect the presence of anxiety and depression in healthcare workers.

Therefore, our study aimed to assess mental health outcomes, levels of anxiety and depression, and factors affecting psychological status in healthcare workers during COVID-19 pandemic in Turkey.

## Material-method

### Procedure

This is a web-based, cross-sectional study performed via an online survey in April 2020.Between April and May 2020, a total of 300 health workers, 193 males and 107 females, participated in the survey. The study was performed nearly one month after the start of COVID-19 outbreak in Turkey. Data was collected through an online questionnaire using SurveyMonkey software (SurveyMonkey, San Mateo, CA, USA). The web-based questionnaire link was sent to the members of Turkey Respiratory Society, including chest disease specialists, pulmonology research assistants and pulmonology related group members (nurses, technicians, physiologists) via e-mail to their personal e-mail addresses. The healthcare workers who joined this survey and answered all the questions were included in the study.

The study was approved by Local Ethic Committee of Gazi University School of Medicine, in accordance to the principles in the Declaration of Helsinki.

### Participants

Health care workers who work in Turkey's seven geographical regions participated in the study.The majority of participants were in the 40–49 age group. There were 246 (82%) doctors, 33 (11%) nurses and 21 (7%) other healthcare professionals as participants in our study. Only 17.3% of healthcare workers had at least one comorbidity. Table 1 shows the sociodemographic features of the participants.

**Table 1:** Demographics of the healthcare workers

Demographics and characteristics		Participants	
		(n=300)	%
**Age**	**20–29**	48	16
	**30–39**	57	29
	**40–49**	107	35.7
	**50–59**	47	15.6
	**>60**	11	3.7
**Gender**	**Male**	107	35.7
	**female**	193	64.3
**Occupation**	**Doctor (specialities)**	242	82
	Pulmonology	200	82.6
	Chest surgery	14	5.8
	Internal medicine	7	2.9
	Infectious disease	5	2.1
	Family medicine	6	2.5
	Emergency medicine	2	0.8
	Other	8	3.3
	**Nurse**	32	10.9
	**Healthcare stuff**	21	7.1
**Type of working** **hospital**	**University**	113	37.6
	**State**	151	50.3
	**Private**	29	9.8
	**Family medicine practice**	2	0.7
	**Health center**	5	1.6
**Comorbidity**	**Present**	52	17.3
	**Not present**	248	82.7

### Measures

The survey included 36 questions with multiple choices. The first 8 questions based on sociodemographic features, such as gender, age and occupation. Questions between 9 and 22 were about COVID-19 experiences (Appendix 1).

The anxiety and depression of the study subjects was evaluated using the hospital anxiety and depression scale (HADS), which included questions from 23 to 36^9^. This scale contains two subscales measuring symptoms of depression (HADS-D) and anxiety (HADS-A). It includes seven statements on each disorder, and each statement is ranked on a 4-point (from zero to three) scale with zero denoting the lowest and three denoting the highest level of anxiety or depression. Aydemir et al. published validity and reliability of Turkish Version of HADS, which had a cut-off score more than 10 for HADS-D and 7 foHADS-A revealing potential depression and anxiety^10^. The Cronbach's alpha values were 0.7426 for HADS-A and 0.6962 for HADS-D in this study^10^. The Cronbach's alpha coefficients for anxiety and depression in our study were 0.81 and 0.79, respectively.

### Statistical analysis

Statistical analyses were performed using SPSS version 20.0®. Descriptive statistics were used to present baseline characteristics. Continuous variables were expressed as mean ± standard deviation and median (range). Chi-square test (χ2) was used to compare the differences between groups. Student t test was used to compare the means of 2 independent samples. A P-value < .05 was considered statistically significant. Participants were grouped as individuals with anxiety and depression based on HADS cut-off scores, and binary logistic regression was used to identify factors associated with anxiety and depression. Odds ratio (OR) and 95% confidence interval (95% CI) were obtained from logistic regression models.

## Results

There were 219 participants (73%) who think that they have sufficient knowledge about Coronavirus. The rate of healthcare professionals who directly followed the coronavirus patient was 73%. The most frequently used protective health equipment was told as the medical mask in contact with the possible / definitive COVID-19 patients (91.2%). Nearly 7% of healthcare workers stated that they received treatment with the diagnosis of COVID-19. While 77.2% of participants expressed feelings of anxiety and fear due to COVID-19, 4% stated that they received psychological support. Table 2 shows the perceptions and experiences of healthcare professionals about COVID-19.

**Table 2:** Perceptions and experiences of healthcare workers about COVID-19

Statement		Yes, n (%)	No, n (%)
I think my knowledge about COVID-19 is sufficient.		219 (73%)	81 (27%)
There has been COVID-19 case in my hospital.		279 (94%)	18 (6%)
There is inpatient service or polyclinic of COVID-19 at my hospital.		258 (96.5%)	9 (3.5%)
I take care of COVID-19 patients.		189 (73%)	70 (27%)
I had contact with the COVID-19 patient without a mask.		213 (82.2%)	46 (17.8%)
I took a swab sample from the COVID-19 patient.		118 (45.6%)	141 (54.4%)
It is a protective health equipment that I use in any case in contact with the COVID-19 patient.	Medical mask	229 (91.2%)	22 (8.8%)
	Medical gloves	207 (82.8%)	43 (17.2%)
	N95 / FFP2 mask	195 (76.8%)	59 (23.2%)
	Shield / protective glasses	183 (72.3%)	70 (27.7%)
	Disposable overalls Apron	185 (74%)	65 (26%)
		219 (86.2%)	35 (13.8%)
I make possible / definitive COVID-19 patients wear medical mask during hospital stay.		257 (99.2%)	2 (0.8%)
I gave a swab sample for COVID-19.		78 (30.2%)	180 (69.8%)
I was treated with the diagnosis of COVID-19.		17 (6.6%)	241 (93.6%)
I totally obey the isolation rules at home.		203 (78.7%)	55 (21.3%)

COVID-19: Coronavirus disease 2019

Participants' mean HADS-A and HADS-D scores were 9.72 ± 5.03 and 9.62 ± 4.32, respectively. HADS total mean score was 19.16 ± 8.84. According to HADS, 44.6% of healthcare workers scored above the anxiety and 68.2% scored above the depression cut-off points. In other words, over two-thirds of healthcare workers were at risk for depression, and nearly half of them had this risk for anxiety.

Female healthcare professionals had statistically significant higher HADS-A and HADS-D scores then male (p<0.001 and p=0.004, respectively). Similarly, participants under 50 years old had significantly higher HADS-A and HADS-D scores when compared with those over 50 (p<0.001 and 0.007, respectively). Mean HADS-A scores were statistically higher in the healthcare workers who who took care of COVID-19 patients (p=0.003) and had taken a swab sample from a patient with SARS-CoV2 (p=0.037). There were statistically significant higher HADS-D score in healthcare workers who had at least one comorbidity (p= 0.022). Mean HADS-A and D scores according to characteristics of participants are in Table 3.

**Table 3:** Mean HADS-A and D according to characteristics of participants

Respondent characteristics		HADS – A (mean ± SD)	St. Error mean	df	p value	HADS -D (mean ± SD)	St. Error mean	df	p value
Age ≥ 50		7.42±4.59	0.648	256	<0.001	8.16±4.05	0.572	256	0.007
Age < 50		10.27±4.99	0.346			9.97±4.32	0.299		
Gender	Male	8.13±4.89	0.379	256	<0.001	8.58±4.06	0.428	256	0.004
	Female	10.57±4.92	0.515			10.18±4.36	0.336		
Occupation	Doctor	9.53±5.00	0.324	256	0.050	9.47±4.27	0.277	256	0.062
	Other health workers	11.95±5.00	1.118			11.35±4.65	1.039		
Status of doctor	Specialist doctor	9.61±5.08	0.362	233	0.741	9.56±4.42	0.315	65.136	0.465
	Resident doctor	9.32±4.56	0.740			9.11±3.32	0.538		
Comorbidity	Present	10.19±5.41	0.825	252	0.520	11.05±4.70	0.717	252	0.022
	Not present	9.64±4.95	0.340			9.39±4.19	0.289		
Knowledge about COVID-19	Sufficient	9.47±4.93	0.400	256	0.179	9.37±4.22	0.442	256	0.111
	Insufficient	10.43±5.30	0.346			10.34±4.54	0.360		
Taking care of COVID-19 patients	Yes	10.24±5.21	0.380	143.343	0.003	9.84±4.38	0.320	254	0.277
	No	8.31±4.38	0.519			9.18±4.07	0.494		
Taking swab sample from COVID-19 patient	Yes	10.45±5.09	0.470	255	0.037	10.13±4.48	0.376	255	0.098
	No	9.14±4.94	0.418			9.24±4.06	0.379		
Having diagnosis of COVID-19	Yes	9.77±4.12	0.338	256	0.178	9.66±4.33	0.340	256	0.716
	No	9.22±4.03	0.412			9.54±4.21	0.388		

COVID-19: Coronavirus disease 2019, HADS-A: Hospital Anxiety and Depression Scale - Anxiety, HADS-D: Hospital Anxiety and Depression Scale – Depression, St: Standard, df: degree of freedom

Anxiety scores were significantly higher among healthcare workers younger than 50 (p = 0.001, compared with those over 50), women (p = 0.008, compared with men), and individuals taking care of COVID-19 patients (p = 0.005, compared with those who did not provide healthcare to COVID-19 patients), Younger participants (<50), female healthcare workers and individuals with chronic diseases had statistically significant higher depression scores (p = 0.040, 0.019 and 0.042, respectively). Table 4 reveals the association between respondent characteristics and psycological status.

**Table 4:** Association between respondent characteristics and presence of anxiety/depression according to HADS

Respondent characteristics		Anxiety (n)	No Anxiety (n)	Value	df	p value	Depression (n)	No Depression (n)	Value	df	p value
**Age ≥ 50**	Yes	12	39	10.626	1	0.001	29	22	4.269	1	0.040
	No	103	104				147	60			
**Gender**	Male	30	60	7.068	1	0.008	53	37	5.547	1	0.019
	Female	85	83				123	45			
**Occupation**	Doctor	94	123	1.662	1	0.283	148	69	1.388	1	0.822
	Other health workers	21	19				28	12			
**Status** **of doctor**	Specialist doctor	80	106	0.085	1	0.905	125	61	1.117	1	0.698
	Resident doctor	15	19				24	10			
**Comorbidity**	Present	94	119	0.097	1	0.756	36	9	3.490	1	0.042
	Not present	21	24				140	73			
**Knowledge** **about** **COVID-19**	Sufficient	81	109	1.101	1	0.294	126	64	1.202	1	0.273
	Insufficient	34	34				50	18			
**Taking care** **of COVID-19** **patients**	Yes	94	94	8.569	1	0.005	130	58	0.052	1	0.704
	No	21	48				46	23			
**Taking a** **swab** **sample**	Yes	58	59	2.023	1	0.155	117	86	2.509	1	0.113
	No	57	83				140	90			
**Having** **diagnosis** **of COVID-19**	Yes	5	12	1.915	1	0.183	11	6	0.061	1	0.710
	No	110	129				165	74			

COVID-19: Coronavirus disease 2019.

Df: degrees of freedom.

Being younger than 50 (OR, 2.675, 95% CI, 1.260–5.678; p = 0.010) and taking care of COVID-19 patients in hospitals (OR, 1.912; 95% CI, 1.051–3.556; p = 0.0046) were independently associated with anxiety risk among healthcare workers. On the other hand, being female (OR, 1.972, 95% CI, 1.073–3.441; p = 0.028), being younger than 50 (OR, 2.247, 95% CI, 1.103–4.577; p = 0.026) and having a comorbidity (OR, 2.718; 95% CI, 1.144–6.459; p =0.024) were predictors for depression among healthcare proffessionals. Table 5 shows the binary logistic regression analysis about the risk factors for depression and anxiety.

## Discussion

This web-based study investigated if mental health of Turkish healthcare workers was negatively affected by COVID-19 outbreak. Our results demonstrated a high risk of anxiety and depression in healthcare professionals. Female gender, younger age (less than 50), having an organic disease or a contact with COVID-19 patients were found as potantial risk factors.

Healthcare professionals seemed to be at high risk of anxiety and depression during COVID-19 outbreak. The 2019 coronvirus disease is a public health problem that causes international concern and negatively affect the pyschological state^11^. Li et al. demonstrated that 24% of healthcare workers had varying levels of anxiety, and 33% of them had depression during COVID-19 pandemic^12^. There was a similar pyschological impact on the public during the 2003 SARS outbreak^13^. This indicates that the pandemic is destabilizing the pyschological status of the whole society affected by COVID-19.

As all medical health workers are first-line fighters during COVID-19 outbreak, they have a higher risk of exposure to the virus than almost everyone. This fact may disrupt the pyschological stability of the healthcare workers. The main reasons for the psychological distress might be related to the many difficulties, such as feeling of being unsafe at work, the long-term workload at hospitals and the lack of medical protective equipment^3^. Our study revealed that 68% of healthcare workers were at risk of depression and 45% of them were at risk of anxiety according to HADS. Our findings about anxiety and depression seem extremely high when compared with similar studies. The overall prevalance of general anxiety disorder and depressive symptoms were 35% and 20% respectively in one study about COVID-19^14^. Lai et al. reported symptoms of depression in 50.4% and anxiety in 44.6% among healthcare workers in Coronavirus outbreak, which is the study with the closest results to our rates of anxiety and depression^15^. There is only one study evaluating the levels of anxiety and depression by HADS score during the COVID-19 pandemic, like ours. Özdin et al. demonstrated anxiety and depression levels and rates of Turkish society as 45.1% and 23.6% respectively (16). Anxiety level seems to be same in both studies. Due to results of both studies, we can say that medical health workers have a greater risk for anxiety and depression during COVID-19 outbreak.

In our study, anxiety and depression levels were found to be higher in women healthcare workers. It may reflect the greater psychiatric impact of COVID-19 pandemic on women. Female gender is vulnerable to depression according to literature data^17^. Many previous researches reported that women were more likely to have anxiety^18^ and depression^17^ than men. This fact may be associated with some hormonal (menstruation, menopausis) and emotional (sensitive and fragile personality) features. Women are also estimated to be more affected than men during pandemics^16^, ^20^. Pandemics may cause female gender to suffer more re-experiencing and negative alterations in cognition and mood^21^. This may explain why women were more severely affected in our study.

Healthcare workers younger than age of 50 were found to have high levels of anxiety and depression in our study. Huang et al. similarly reported that younger participants (< 35 years) were more likely to develop anxiety and depressive symptoms during COVID-19 outbreak^14^. These study findings suggest that younger healthcare workers were more vulnerable and cope less with the chaotic mood created by COVID-19 outbreak. According to binary logistic regression analysis, we found that presence of at least one comorbidity and taking care of COVID-19 patients may be potantial risk factors for depression and anxiety, respectively. Having an organic disease and having a contact with COVID-19 patients in hospitals were specified as common and independent risk factors for anxiety and depression among medical health workers in a previous study^22^. It is understandable that those with comorbidity or those who followed COVID-19 patient may have higher depression or anxiety levels.

Ministry of Health have made some arrangements for healthcare workers in Turkey who are on duty during COVID-19 pandemic at the beginning of outbreak. However, due to high anxiety and depression levels detected in our study, we think that some additional precautions should be taken. Providing the best physical, mental, and social conditions may increase confidence and morale of our healthcare workers^3^. First of all, professional psychological support interventions should be directed to healthcare workers, working directly with COVID-19 patients. The most evidence-based treatment, cognitive behaviour therapy may help to prevent the spread of infection during the pandemic^23^.

Another important topic is preventive measures, such as personal protective equipment. Strengthening prevention and control measures would bring potential psychological benefits^21^. Wang et al. demonstrated that participants who did not wear face masks had significantly higher levels of anxiety, depression and stress^24^. Personal psychoneuroimmunity prevention measures including hand hygiene and wearing face masks, which were found to be associated with less severe psychiatric symptoms^25^, may be an important precaution. Also, healthcare workers should be warned about subjects such as regular diet and adequate sleep time during pandemic.

This study has some limitations. First, the study has a cross-sectional design, which does not allow to make causal inferences. As the survey process included peak time (end of April) of the pandemic in Turkey, this may cause the high level of anxiety and depression among participants. Second, we had no chance to assess the individuals' psychological conditions before COVID-19 outbreak. Third, our study mainly used self-reported questionnaires to measure psychiatric symptoms and did not make clinical diagnosis. A study involving structured clinical interview and functional neuroimaging as the gold standard for establishing psychiatric diagnosiswould give more realistic results.

## Conclusion

Healthcare professionals were at high risk of anxiety and depression during COVID-19 outbreak. Our report demonstrated potential risk factors as female gender, younger age (less than 50), having a comorbidity and having a contact with COVID-19 patients for healthcare workers to develop anxiety and depression. Therefore, these groups may have priority in terms of psychological support.
